# Collective imaginaries of caring landscapes for rural youth: a concept mapping study in northern Sweden

**DOI:** 10.1186/s12889-021-12223-4

**Published:** 2021-11-30

**Authors:** Frida Jonsson, Monica Christianson, Maria Wiklund, Anna-Karin Hurtig, Isabel Goicolea

**Affiliations:** 1 Department of Epidemiology and Global Health, Umeå University, Umeå, Sweden; 2 Arctic Research Centre (Arcum) at Umeå University, Umeå, Sweden; 3 Department of Nursing, Umeå University, Umeå, Sweden; 4 Department of Community Medicine and Rehabilitation, Unit of Physiotherapy, Umeå University, Umeå, Sweden

**Keywords:** Northern Sweden, Rural, Youth, Landscapes of care, Utopia as method, Concept mapping

## Abstract

**Background:**

In the current study, the approach of ‘utopia as method’ was combined with the concept ‘landscapes of care’ to explore collective imaginaries of caring landscapes in relation to young people living in rural northern Sweden, while focusing specifically on what such landscapes should ideally look like, and how various strategies could help to realise the visions.

**Methods:**

The research was conducted using a modified concept mapping methodology comprising three phases of data collection and analysis. This facilitated the integration of tacit knowledge and utopian visions of young people, professionals and policymakers living and working in various parts of northern Sweden.

**Results:**

The results indicated that caring landscapes should: ‘provide services responsive to young people’s wishes and needs’, ‘be organised around values of safety, equity and youth participation’, and ‘rework metro-centredness’ in order to care *for*, *with* and *about* rural youth.

**Conclusions:**

The findings can be viewed as an imaginary reconstitution of communities in rural northern Sweden, but also as hypothetical building blocks to be used for developing caring landscapes and a ‘good countryside’ where young people have the possibility to live a good life in decent health.

**Supplementary Information:**

The online version contains supplementary material available at 10.1186/s12889-021-12223-4.

## Background

*“What might constitute appropriate visions for rural futures, of our imaginaries of rural places into the twenty-first century?”* [1]

Using Levitas’ [2] approach of ‘utopia as method’ and by referring to desired alternatives for rural futures, Shucksmith [1] encourages us to imagine and discuss not only what might constitute the good city [3], but also what a ‘good countryside’ look like. Building on these concepts, while integrating Milligan and Willes’ [4] framework of ‘landscapes of care’, this study aimed to explore collective imaginaries of caring landscapes in northern Sweden, specifically in relation to rural youth.

Existing research in northern Sweden – an area depicted as sparsely populated, comprising 60% of the Swedish land area and only 12% of the population [5] – has indicated that a good life in decent health for rural youth may be contingent upon notions of care, which involve experiences bound up in dynamic and multi-layered landscapes [6–10]. Our preceding work has further depicted how feeling belongingness to, and at home in, one’s locale coupled with opportunities to participate in local decision-making while having access to practical and emotional support, characterise landscapes of care for rural youth [10]. However, by also bringing negative attributes to the fore through the concept of despair, the results simultaneously illustrated how rural settings may be largely limited to adult expectations and institutions [11, 12]. This, in turn, seemed to have an adverse influence on young people’s health and wellbeing, for example, due to gaps in service delivery, lack of leisure options and subsequent feelings of frustration, disappointment and alienation [10].

The ‘despair’ of rural communities is not only a discursive representation, but has to be understood in relation to political and economic trends. In this regard, the introduction of market- and urban-oriented reforms in many European countries, including Sweden, has typically contributed to a retrenchment of welfare resources in rural communities [13]. By being linked to a subsequent portrayal of ‘rurality’ as something inferior and in deficit, the decline of rural welfare has shaped the life trajectories of many young people. More specifically, during the last few decades, rural areas of the global north have experienced substantial out-migration, especially of young adult women [14]. This has contributed to a view of young people as a precariously connected group who will ultimately abandon their childhood homes [15]. However, although attracting and retaining young people has been considered essential for the survival of these communities, those who stay are typically looked upon and/or perceive themselves as failures because remaining in ‘the rural’ usually does not align with norms of personal success [6, 7, 16, 17].

Within much of youth studies to date, a metropolitan focus has contributed to a lack of attention being paid to the lives and lived experiences of rural youth; to representations of youth sub-cultures as being mainly an urban phenomenon; and to the construction of rural spaces as old-fashioned, traditional, passive and occasionally boring or ‘dull’ [18–21]. These images, which portray rural places as ageing and less youthful or ‘cool’, further align with views typically depicted in politics, academia, the media and popular culture [22–26]; while also corresponding with the tendency to direct services and programmes in these areas towards the elderly [27].

As notable exceptions to the above negative foci, and as a slight nuance to the simplistic description of rural areas as unattractive to, and almost uninhabitable for, youth, studies have emphasised how young people’s relationships with their locale incorporate not only abhorrence and rejection, but feelings of longing and belonging [26, 28]. In addition, while their experiences and expressions seem to be at least partly contingent upon broader representations of a ‘rural idyll’, young people’s attachment to, and identification with, their birthplace appear to be strongly reflected in senses of harmony, admiration and appreciation [9, 29].

While divisions between urban and rural, city and countryside might be problematic, comparisons with a normative metropolis still exist, and imply a risk of undermining what is meant by, and needed in, more ‘peripheral’ areas. Studies that can inform action by focusing on challenges to, *and* opportunities for, rural areas thus remain crucial [1], especially considering the separate policies through which many countries govern rural places [21]. Building on these notions, the current study will contribute to the literature by ‘recalibrating’ an adult-centric focus by incorporating the diverse voices of young people in the production of rural knowledge [16, 30]. Against this backdrop, the aim of this study was to explore collective imaginaries of caring landscapes in relation to young people living in rural northern Sweden, while focusing on what such landscapes should ideally look like, and how various strategies could help to realise these visions.

## Conceptual framework

The current study builds upon and integrates two main perspectives: the concept of ‘landscapes of care’ as described by Milligan and Wiles [4] and Levitas’ [2] ‘utopia as method’ – where the latter will be considered in relation to Shucksmith’s [1] discussion of what might be appropriate visions for rural futures and a ‘good countryside’.

Following Milligan and Wiles [4] and our previous research [10], in this study ‘landscapes of care’ is understood as a multi-layered framework, with the experiences, provisions and practices of care being seen as shaped by acts and affective dimensions at interpersonal levels; arrangements and accountabilities at organisational levels; and policies, discourses and norms at structural levels. As reflected in the adjacent concept of ‘therapeutic landscapes’, these processes are then presumed to take place within, and across, landscapes that constitute symbolic spaces of familiarity and culture, while being characterised by physical features and social conditions [31]. More specifically, in this study, care is seen as a having a dual meaning referring to the practical and relational work involved in the provision of different forms of support as well as to a personal quality of tending for others in response to their needs [32]. Based on this notion, we draw upon the concepts of caring *for* and caring *about* as depicted by Milligan and Wiles [4] to acknowledge this distinction, and to differentiate between activities and actions involved in ‘care-giving’ and affective elements involved in ‘being caring’, respectively.

To explore collective imaginaries of caring landscapes, we integrate Levitas’ [2] concept of ‘utopia as method’. Following Bloch [33], Levitas [2] defines utopia as “the expression of the desire for a better way of living or of being” [2 p. 20]. Rather than approaching the concept in a conclusive way as an end in itself, she suggests that it is understood – and indeed used – as a basis to imagine, articulate and translate ideas about and hopes for a reconstitution of society into hypothetical, albeit more specific institutional terms. In this regard, Levitas [34] recognises the reflexivity, provisionality and fallibility of utopia while emphasising its transformative potential, which “enables us to think about where we want to get to, and how to get there from here” [34 p. 300]. However, at the same time, she agrees with Bloch [33] that opportunities to imagine alternative social systems are not equally shared, but shaped by the power and positions of different groups [35].

Building on the architectural mode of ‘utopia as method’ [2, 35], which implies that the imagining of possible future scenarios should not be limited to abstract concepts but concrete in terms of principles and structures that would help to realise them, Shucksmith [1] has initiated a discussion about what might be appropriate visions for rural futures and a ‘good countryside’. In this regard, he draws upon the concept of ‘place shaping’ to illustrate how imaginaries of desired alternatives can be constructed in relation to specific landscapes. However, defined as “self-conscious collective efforts to re-imagine a city, urban region or wider territory and to translate the result into priorities (...)” [36 p. 46], the concept of ‘place shaping’ appears to be largely metro-centric. In this sense, Shucksmith’s [1] work is a critique, not only of an extensive body of rural literature that has focused on the sustainability of peripheral communities rather than on the underpinnings of a ‘good countryside’, but also of the vast number of studies that have explored what constitutes the good city without considering what its rural counterpart might look like. To challenge these knowledge gaps, Shucksmith [1] translates Amin’s [3] four ethical elements of solidarity from the urban to the rural context, while concluding that a ‘good countryside’ is (or should be) “socially inclusive, networked rather than insular; agentic rather than passive; reflexive and resilient; with the support of an enabling state” [1].

To this end, although both Levitas and Shucksmith stress the importance of considering who gets to imagine alternative societal futures, the absence of youth perspectives in their own and related writings, passes largely unnoticed. In the current study, we seek to bridge this knowledge gap by integrating the conceptual work of Milligan and Wiles [4] with a modified concept mapping methodology [37] to explore collective imaginaries of caring landscapes in relation to young people living in rural northern Sweden.

## Methods

### Setting

This study is situated in the northernmost part of Sweden, popularly called ‘Norrland’. With approximately 4.8 residents per square kilometre, this is a sparsely populated region where people are dispersed across small rural villages in the northwest inland or concentrated in somewhat larger cities along the southeast coast. This area is home to the Sámi population, comprising about 20–40,000 individuals [38] as well as a large number of international migrants, of whom many are unaccompanied children and youth [39]. In this study, our focus is directed specifically towards Norrland’s ‘resource peripheral’ interior, which comprises areas that are similar, albeit not identical, to each other in demographic and socioeconomic structure. These areas are typically characterised by depopulation and low proportions of inhabitants with a university degree while being dominated by traditional rural sectors such as agriculture, forestry, mining and tourism [40]. In certain parts of Norrland, social activism against the withdrawal of public services (mainly healthcare) has also flourished (Enlund [13]).

### Methodological approach

To address the aim and integrate the tacit knowledge of young people, professionals and policymakers, we used concept mapping as the overarching methodology [37]. By giving participants the opportunity to visualise their ideas, to discuss an issue of mutual interest, and to contribute knowledge valuable to develop culturally tailored health interventions, this approach has been considered useful to increase community involvement in research [41]. In addition, since this methodology allow participants to identify answers to a ‘prompt’ or a ‘focused’ question that is often visionary, aiming for advancements or improvements [42], the approach also has the potential to function as a catalyst for imagining alternative scenarios or futures.

As explained by Kane and Trochim [37], concept mapping involves the generation and integration of both qualitative and quantitative data by participants in sequential steps. The process begins with the generation of ideas (through brainstorming), followed by the structuring of these ideas (through sorting and rating), and ends with the development of ‘conceptual maps’ (through the application of multivariate statistical methods). Our approach differed from this procedure in two major ways. Firstly, compared to the more ‘traditional’ approach [37], as researchers we took a more active role in the process of analysing the data. In particular, we recognised ourselves as actively co-producing the results in dialogue with the participants, rather than merely supporting them to visualise and report their ideas. Secondly, we placed the strongest emphasis on the qualitative aspects of the process; consequently, our findings and discussion build not only on one final map based on multivariate statistical methods, but on findings from three phases of data collection and analysis. In the following, we describe the specificities of each phase (see Table 1 for an overview).

**Table 1:** Overview of the study design and different phases of research

	Phase one	Phase two	Phase three
**Time period**	August – September 2019	October – November 2019	December 2019 – January 2020
**Participants**	*Young people* 63 (29 women, 33 men, 1 not either or)	*Young people* 6 (4 women, 2 men)	*Young people* 3 (all women)
	*Professionals* 44 (33 women, 11 men)	*Professionals* 25 (19 women, 6 men)	*Professionals* 16 (11 women, 5 men)
**Methodological approach**	*‘Secondary’ analysis* Researchers re-analysed qualitative data collected during previous research.	*Workshop* Participants brainstormed to identify strategies.	*Concept mapping* Participants sorted and rated the identified strategies.
**Analytical approach**	*Qualitative* The researchers’ analysed the transcribed and previously collected interviews to identify strategies and developed five thematic clusters of strategies.	*Qualitative* The researchers’ synthesised strategies identified in the workshop and developed a conceptual map with six thematic clusters of strategies.	*Mixed methodology* The researchers’ used multivariate statistical methods to summarise the identified strategies and develop a conceptual map illustrating five clusters of strategies.

### Study design

#### Phase one – ‘secondary’ analysis

In the first phase, a ‘secondary’ analysis [43] of qualitative data collected for another study conducted by the research team [10] was carried out to explore collective imaginaries of caring landscapes and strategies that could help to realise the visions. The data gathered for this previous study provided enough material for a follow-up study by including 42 interviews in total: 16 individual ones conducted with professionals and 26 FGDs, of which 11 were with professionals and 15 with young people. Furthermore, it captured different youth voices regarding ethnicity/‘racialization’, gender, functionality and sexuality; areas that varied in location, size and socio-economic situation; and professionals working across sectors such as health centres, school health, integration units, youth clinics, specialised psychiatric care and youth clubs. During the interviews (which were digitally recorded and transcribed verbatim), participants were asked to reflect upon aspects related to youth, rurality, health, wellbeing and access to services as well as to give suggestions for improving or strengthening the health and/or social situation for young people.

Using this data to investigate questions beyond those for which it was originally intended [43], the analysis involved reading and re-reading the transcripts, including systematic markings of aspects that reflected imaginaries of caring landscapes and strategies that could help to realise these visions. Each interview was then inductively coded and thereafter codes with similar content were grouped together, following Braun and Clarke’s [44] analytical approach. From this procedure, two authors (FJ and IG) developed five thematic clusters of codes that were discussed and agreed upon by the research team.

#### Phase two – participatory workshop

In the second phase, the visions and ideas of young people, professionals and policymakers who participated in a one-day workshop in November 2019 was used to explore collective imaginaries of caring landscapes and strategies that could help to realise the visions. In total, 31 participants living and working in various parts of northern Sweden attended the workshop: 6 young people and 25 professionals/policymakers working with youth- and/or health-related issues at municipal or regional levels (such as school health, youth clinics, public health units and social services). After being informed about the research project as a whole [see 27], the participants were divided into five groups of 6-7 participants. One researcher from the team acted as a moderator in each group. During the group work, participants were asked to envision an ideal caring landscape, and to think of possible answers to the ‘prompt’ statement: *in order to create opportunities for rural youth to live a good life in decent health, the strategies or efforts needed are...* They were then asked to write down their individual answers on separate post-its and to share them with the rest of the group. Together, they were then asked to collectively organise all of the strategies into clusters in a way that appeared meaningful to them by placing and grouping them all on a big sheet of paper, and if possible, putting a label to each cluster of strategies.

After the workshop, two authors (FJ and IG) collected all five sheets of paper containing the strategies and cluster labels, translated them into English and then analysed and merged together separate strategies or clusters of strategies from the different groups. Here, the aim was to include as many perspectives as possible. This meant that we proceeded by conflating clusters when they coincided, expanding clusters with statements from several groups and including separate clusters, even if they only appeared in one of the groups. Based on this process, six thematic clusters of strategies were developed and then discussed and agreed upon by the whole research team.

#### Phase three – ‘traditional’ concept mapping

In the third phase, a conceptual map was developed following the more ‘traditional’ concept mapping approach [37] to explore collective imaginaries of caring landscapes and strategies that could help to realise the visions. This implied that all 31 workshop participants and 11 additional individuals who had been unable to attend (1 young person and 10 professionals), were invited to participate in a sorting activity conducted individually. To facilitate this process, a consolidated list of 68 unique strategies was first developed by two authors (FJ and IG). This involved: i) compiling strategies from phases one and two, ii) adding new ones provided by individuals who could not join the workshop, and iii) refining the data by removing duplicates and merging strategies that were similar in character or content. Based on this material, an online questionnaire was designed using the ‘GroupWisdom’ software [45]. The participants were then asked to engage in the web-based sorting activity by grouping strategies in a way that appeared meaningful to them. Out of the 42 individuals who were invited, 19 completed the questionnaire – 16 professionals and 3 young people.

Using the ‘GroupWisdom’ software, information underlying the conceptual map was gathered and used in the following steps. Firstly, the sorted data was analysed using multi-dimensional scaling to generate point maps. This meant that strategies were plotted based on the number of times participants grouped them together, with those that were frequently put together positioned close to each other. Hierarchical cluster analysis was then applied to generate cluster maps, which implied that strategies were aggregated into clusters based on their proximity to each other on the point map [37]. Ratings based on *relevance* and *feasibility* on a 5-point Likert-type scale were also calculated for each strategy. Maps depicting how strategies were grouped including solutions ranging from 4 to 8 clusters were evaluated, and with the most appropriate number of clusters being determined through discussion within the research team. Successive levels of clustering were evaluated based on their conceptual coherence and the value of precision offered at each level. Based on this process, a conceptual map was developed comprising five clusters of strategies.

## Results

In this section, we briefly present the results from phases one and two to then concentrate on the concept map developed in phase three, which partially builds upon findings from the two previous phases.

From the *first phase*, we developed five themes that captured the collective imaginaries and clusters of strategies that could help to realise the visions. These themes indicated that caring landscapes should: 1) ‘*provide youth-friendly health services*’, which implied a need for continuous, holistic, equitable, coordinated and flexible care for young people involving access to preventive and curative initiatives; 2) ‘*create options and opportunities*’, which implied the availability of, and access to, leisure activities, education and employment to ensure that all young people would have the opportunity to ‘follow their dreams’; 3) ‘*be inclusive and open*’, which implied that young people should be able to feel safe, a sense of belonging and be respected for who they are; 4) ‘*ensure meaningful youth participation and influence*’, which implied that young people should have the opportunity to shape decision-making beyond consultation or utilitarian engagement; and 5) ‘actively resist metro-centric structures’, which called for changes to the growing concentration of power and resources in cities and urban areas. Supplementary Table 1 provides examples of strategies included in each theme.

From the *second phase*, we developed six themes that captured collective imaginaries of caring landscapes and clusters of strategies. According to the participants’ utopian visions, such landscapes should: 1) ‘*offer a future*’, in terms of housing and job prospects, good educational environments, spaces for meaningful leisure and accessible healthcare for rural youth; 2) ‘*facilitate good collaboration*’, focusing on communication and coordination between services with responsibilities for rural youth; 3) ‘*ensure connectedness*’, emphasising the importance of internet connections and transportation systems for rural youth; 4) ‘*provide a safe and stimulating environment*’, in terms of physical and virtual spaces where young people interact (such as parks, schools, sports clubs and the internet); 5) ‘*ensure that (all) youth have the power to influence*’, stressing the importance of creating an inclusive climate that supports young people, that offers ‘second chances’ and that promotes participation in decision-making; and 6) ‘*redress urban-centred images and policies*’, in terms of broader political decisions related to privatisation and centralisation, as well as the need to create and maintain a positive image of ‘the rural’. Supplementary Table 2 provides examples of strategies included in each theme.

**Fig. 1:**
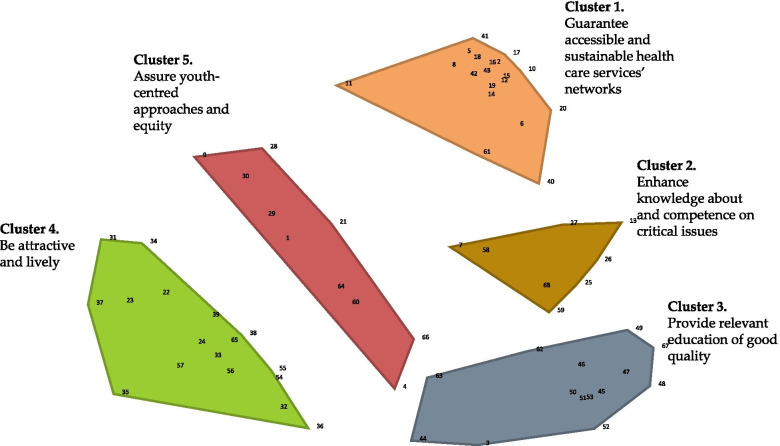
Conceptual map developed during phase three comprising five clusters of strategies that depict central aspects of caring landscapes

From the *third phase*, a conceptual map (see Fig. 1) was developed comprising five clusters of strategies that depicted collective imaginaries of caring landscapes. The first cluster ‘*guarantee accessible and sustainable healthcare service networks*’, focused on strengthening existing services (by reducing waiting times and offering better information), on providing a wider array of relevant services (such as youth clinics), on finding alternative solutions to promote access to services (using eHealth or mobile teams), and on approaches that could ensure that young people’s problems are ‘taken seriously’. The second cluster ‘*enhance knowledge about and competence on critical issues*’, focused on the need to discuss questions that are crucial for the diverse group of rural youth such as LGBTQI+, gender norms, Sámi culture, gender-based violence, ableism and mental ill health. The third cluster ‘*provide relevant education of good quality*’, focused on the importance of improving the school system in rural areas, for example, by increasing the number of (qualified) teachers, by facilitating educational continuity (through support for those who drop out of school or move away to continue their education), by ensuring that the school setting is a safe space (by preventing harassment and bullying), by strengthening school health services, and by working more actively with health-related questions (such as sex education, drug and alcohol prevention, mental ill health). The fourth cluster ‘*be attractive and lively*’, focused on aspects that could make rural places more interesting for young people, including improvements in infrastructure, cultural life, employment possibilities, leisure and democratic participation. The fifth and final cluster ‘*assure youth-centred approaches and equity*’, focused on inclusiveness, for example, by acknowledging the diversity of young people and by implementing the Child Convention. Supplementary Table 3 provides examples of strategies in each cluster as well as their average ratings on the relevance and feasibility scales.

## Discussion

Based on the above findings, and by integrating the architectural mode of ‘utopia as method’ [2, 35] with the concept ‘landscapes of care’ where acts or activities of ‘care-giving’ (caring *for*) are differentiated from affective aspects of ‘being caring’ (caring *about*) [4], the following discussion is organised into three conceptual areas, which suggest that caring landscapes should: ‘*provide services responsive to young people’s wishes and needs*’, ‘*be organised around values of safety, equity and youth participation*’, and ‘*rework metro-centredness*’.

### Provide services responsive to young people’s wishes and needs

According to the participants’ collective imaginaries and utopian visions, landscapes that are caring *for* rural youth need to provide them with health care services that are relevant and accessible, formal education that is continuous and sufficiently staffed, leisure activities that give them something meaningful to do as well as opportunities to live and work in their locale.

The numerous strategies proposed to improve access to, and availability of, (mental) healthcare and continuity of formal education, can be seen as a partial response to the dismantling of welfare services that has been ongoing in rural northern Sweden during the last several decades [13]. This focus on healthcare and education may also be expected following the construction of youth as a transitional period where young people are presumed to mature into adulthood and gain appropriate competences [19], while also being ‘at risk’ of both socioeconomic disadvantage [17] and various health problems [46]. However, in parallel with these notions, concerns about the lack of housing and work can be seen as a response to considerations about young people’s concurrent wellbeing and their ability to decide for themselves where and how to live their lives. This latter idea further resonates with the participants focus on creating a meaningful leisure to ensure that young people are cared *for* in rural areas.

Adding to the value of offering various services, the strategies proposed by the participants illustrated that their mere existence is not going to guarantee that young people will benefit from them. Instead, following Shucksmith’s [1] reasoning about a networked countryside (which focus on local capacity building by linking institutions at a variety of scales), caring landscapes should comprise a system of collaboration and coordination to ensure that young people are properly cared *for.* As a complementary example of *how* services should be provided to young people by responding adequately and attentively to their wishes and needs, the participants’ visions painted a picture of landscapes that also care *about* young people. This aspect came across in strategies emphasising the importance of having adult professionals who ‘take young people’s problems seriously’ by being committed and compassionate. However, in line with discourses on the agentic countryside [1] and the ‘hardy’ or ‘self-sufficient’ northern Swede [22], this focus on caring *about* seemed to appear, in part, as a way to compensate for workforce shortages and financial constraints [see 47, 48].

Following the participants’ collective imaginaries, the suggestions so far can be seen as *describing* a catalogue of ideal aspirations to be fulfilled, but also as *prescribing* a standardised future for rural youth; one in which the proposed aspects of education, employment and leisure are what (all) young people should strive for and dream about. In line with Levitas’ [2] discussion about the normative dimensions of utopia, the caring landscapes envisaged may be a response to representations of how rural youth should be or behave in order to comply with norms of individual success (i.e. remaining healthy, studying, having a home and a job while engaging in meaningful leisure); and while such ideals might create opportunities for some young people, they may be further excluding others. How to propose utopian visions of caring landscapes that do not exclude certain groups of young people is further analysed in the next section.

### Be organised around values of safety, equity and youth participation

Beyond the provision of services, the participants’ collective imaginaries demonstrated that caring landscapes should be centred on three core values: *safety*, *equity* and *youth participation*. With reference to the first value, emphasis was placed, for example, on the need to prevent bullying and harassment to ensure that spaces where young people interact are safe (such as youth clubs and the internet). This notion, which demonstrates that safety is something that caring landscapes should strive for and/or uphold, can be interpreted as a response to the representation of rural life as ‘safe and good’, where the visibility in and solidarity of small communities contributes to senses of security [see 49]. However, the focus on safety may also be seen as a way to overcome intrusive aspects of informal social control, where gossip and rumours tend to constrain rural youth – especially girls and young women – pressuring them to live and act according to expectations, regardless of their own preferences and interests [20, 50].

Resembling Shucksmith’s conception of a ‘good countryside’ that is socially inclusive by “enabling all to shape and to enjoy rural life” [1 p. 169], the participants also raised several aspects related to *equity.* In particular, by highlighting the need to discuss sensitive or controversial topics and ensure cultural competence among professionals (for example, on the Sámi), their visions illustrated how caring landscapes should allow all young people – irrespective of ethnicity/‘race’, sexuality, gender, health status, functional ability or class – to feel a sense of belonging to or ‘at home’ in their community [see 51]. On the one hand, this vision could be a reaction to the social homogeneity of peripheral locales while revealing a longing for rural life(styles) to become more diverse. On the other hand, it may reflect the stereotypical representation of rural places as less ‘progressive’ and more racist, sexist and homophobic, than urban metropolises [21].

With reference to the last value of *youth participation*, the proposed strategies indicated that caring landscapes should ensure that young people have the opportunity to engage in and influence decision-making beyond mere consultations while receiving a delegated responsibility that comes with both resources and power [see also 52]. Realising this value demanded not only a strengthening of spaces for formalised participation (such as youth delegations), but also a change in how adults interacted with and approached young people (for example, by *really* listening and treating them as equals). So far, our conceptualisation of caring landscapes focusing on service provision above broadly aligns with Milligan and Wiles’ [4] construction of caring *for*, while the values of safety and equity can be seen as a societal or structural version of caring *about*. Building on the participants’ concern with young people’s opportunities to influence these landscapes, we argue that the value of youth participation introduces a new concept: the caring *with*. From this perspective, landscapes that are caring *with* rural youth are those that consider young people as active agents who are involved in the multidirectional and mutually dependent co-production of care [see 32].

### Reworking metro-centredness

While the two previous conceptual areas build upon collective imaginaries and subsequent strategies for how to ensure that landscapes are caring *for*, *about* and *with* rural youth, this last one captures discursive and structural aspects that need to be addressed in order to fulfil utopian visions that appear to be largely contingent upon a felt rural deficit and decline.

Overall, the participants considered opportunities for mobility and connectedness to be vital because such aspects would allow young people to extend their networks beyond the boundaries of rural areas to interact with and gain experience of other people and places. This concern not only questions a strict urban–rural divide, but shows how the participants valued the possibility of being able to transition in and out of their peripheral home. More specifically, in line with research that recognises the dynamic nature of rural youth’s (im)mobilities [see, for example, 6, 7, 15, 30, 53], the participants felt that young people should be able to leave, explore *and* return, but – more importantly – to have a good life in decent health for as long as they remain in ‘the rural’.

Adding to the above focus on creating or sustaining landscapes that remain attractive to, and habitable for, rural youth, stressed was also the need to address the ‘place-based inequality’ [see 54] that largely disfavours peripheral areas of northern Sweden [55]. With regard to this gap, the participants discussed various aspects that should be changed. At a discursive level, and in accord with previous research [22–26], the general view of rural areas as inferior to the urban metropolis was seen as a problem. From a policy perspective, in turn, the participants’ visions followed views similarly portrayed by Enlund [13] in considering the depletion of resources and dismantling of services as major obstacles to improving the situation of rural communities.

However, in contrast to Shucksmith’s [1] view of a ‘good countryside’ that is supported by an enabling state, which extends beyond ideas about “self-help or an invisible hand” [1], the participants generally saw pragmatic and individualised solutions in complex structural issues. This connects with the idea of *reworking,* which implies that people often find focused responses to cope with, rather than to challenge, changing social and economic conditions [56]. In other words, instead of finding answers to the problems of welfare retrenchment and restructuring in the withdrawal of market-oriented reforms [57], the strategies included reminiscences of ‘hardy’ professionals that compensate for a general lack of means and material. Following from ideas that metro-centric governments cannot be trusted to provide sufficient resources [see also 13], this issue could be seen, for example, in strategies suggesting that professionals should ‘be committed and go one step beyond’ to help young people.

### Methodological considerations

In this research, we used a methodological approach comprising three phases of data collection and analysis. Specifically, by adopting concept mapping as an overarching framework, we integrated secondary data from a rich interview study [10] with information that was generated during a one-day workshop and then sorted remotely by the participants using mixed methods. To increase the credibility of our findings, the appendices provide examples of the strategies underlying our analyses.

Notwithstanding these strengths, there are some limitations to our work. The mixture of different data sources and analytical methods means that we have been unable to provide detailed result descriptions of phases one or two, and also that we might have lost theoretical depth in the conceptual map from phase three. However, by conducting our research in three steps and combining the findings from these, we have been able to approach the topic from various perspectives – something that allowed us to better capture the visionary picture that we aimed for. In addition, our goal was to prioritise the perceptions and experiences of youth, and during the first phase we managed to engage a good number of young people from diverse backgrounds. However, in the workshop and sorting activity, only a few young people participated. Alternative ways of interacting with young people in these processes should thus be explored in the future.

## Concluding remarks

In order to create opportunities for rural youth to live a good life in decent health, we – as a society – need to ensure that landscapes are caring *for*, *with* and *about* them. Within such landscapes, services related to healthcare, education, leisure, housing and employment should be provided in a sustainable and coordinated way by adult professionals who acknowledge young people’s capabilities while responding seriously and attentively to their problems. Adding to this notion, which highlights the need to prioritise young people and increase the resources available to this demographic segment in rural peripheries, caring landscapes should be centred on, and guided by, the values of safety, equity and youth participation. This follows from the fact that all young people – irrespective of class, health status, gender, ethnicity/‘race’, sexual identity and/or functional abilities – should be able to feel a sense of security and belonging while simultaneously having the power and opportunities to influence aspects of their lives that matter to them. With reference to structural aspects that extend beyond particular landscapes, while strongly affecting their ability to care for, with and about rural youth, it is important to redress metro-centric discourses and current geographical inequalities that largely disfavour peripheral communities. However, in this process, young people need be seen as active agents, with the ability to choose how to live their lives and the opportunity to be connected to places and people beyond their rural home.

To this end, we conclude following Levitas [34] in stressing that the three conceptual areas can be viewed as an imaginary reconstitution of communities in peripheral northern Sweden. However, with wider engagement, dialogue and responsibility, these can also be seen as building blocks that can make landscapes more caring and contribute to a ‘good countryside’ where young people are able to live a good life in decent health.

## Supplementary Information

**Additional file 1: Supplementary Table 1.** Examples of strategies included in each theme from phase one. **Supplementary Table 2.** Examples of strategies included in each theme from phase two. **Supplementary Table 3.** Examples of strategies included in each cluster from phase three with mean scores on the feasibility (f) and importance (i) ratings.

## Data Availability

The dataset analysed during the current study is not publicly available because it contains sensitive information, but it is available from the corresponding author on reasonable request.
