# Supplementary material for: Collective imaginaries of caring landscapes for rural youth: a concept mapping study in northern Sweden
Source: BMC Public Health. 2021 Nov 30;21:2191. doi: 10.1186/s12889-021-12223-4 (PMC8638169; doi:10.1186/s12889-021-12223-4)
Supplement: Supplementary file 1 — Additional file 1: Supplementary Table 1. Examples of strategies included in each theme from phase one. Supplementary Table 2. Examples of strategies included in each theme from phase two. Supplementary Table 3. Examples of strategies included in each cluster from phase three with mean scores on the feasibility (f) and importance (i) ratings. [file 12889_2021_12223_MOESM1_ESM.docx]

# Collective imaginaries of caring landscapes for rural youth: A concept mapping study in northern Sweden

1. **Frida Jonsson*** (Corresponding author)

**Affiliation 1:** Department of Epidemiology and Global Health, Umeå University, Umeå, Sweden

**Affiliation 2:** Arctic Research Centre (Arcum) at Umeå University, Umeå, Sweden.

**Email:** frida.jonsson@umu.se

**Phone:** +46 (0)90 786 95 97

**Adress:** 5B, Målpunkt P, Försörjningsvägen 7 B, Norrlands Universitetssjukhus, Epidemiologi och global hälsa, Umeå Universitet, 901 85 Umeå

**ORCID:** 0000-0002-5902-3798

1. **Monica Christianson**

**Affiliation:** Department of Nursing, Umeå University, Umeå, Sweden

**ORCID:** 0000-0003-1003-1655

1. **Maria Wiklund**

**Affiliation 1:** Department of Community Medicine and Rehabilitation, Unit of Physiotherapy, Umeå University, Umeå, Sweden.

**Affiliation 2:** Arctic Research Centre (Arcum) at Umeå University, Umeå, Sweden.

**ORCID:** 0000-0001-6636-9597

1. **Anna-Karin Hurtig**

**Affiliation:** Department of Epidemiology and Global Health, Umeå University, Umeå, Sweden

**ORCID:** 0000-0001-7087-1467

1. **Isabel Goicolea**

**Affiliation:** Department of Epidemiology and Global Health, Umeå University, Umeå, Sweden

**ORCID:** 0000-0002-8114-4705

# Supplementary material

**Supplementary table 1. Examples of strategies included in each theme from phase one**

| **Provide youth-friendly  health services** | **Create options and opportunities** | **Be inclusive and open** | **Ensure meaningful youth participation and influence** | **Actively resist metrocentric structures** |
| --- | --- | --- | --- | --- |
| - Drop-in, allow for spontaneous visits - Youth clinic, at a central and independent location - Do more than what is expected, seeing the whole person - Help young people navigate within the health system - Develop and increase the usage of eHealth - Give young people extra time during consultations - Extended telephone hours - Mandatory health checks with the social counsellor - Inform youth about privacy guidelines and how they work | - Leisure activities besides sports - Accessible and safe youth centre - Culturally adapted and flexible employment - Strengthen the rural brand and attractiveness - Highlight and discuss the benefits of living in rural areas | - Theatre that discuss sensitive topics - Talk about masculinity norms - Better education about Sámi in schools - Provide community information to immigrant youth | - Treat young people as equals - Develop youth delegations - Inform and help youth write citizen proposals | - Forum and structures for tighter collaboration - Clarify the roles and responsibilities of actors - Organizations and institutions working with youth organized under the same administration |

**Supplementary table 2. Examples of strategies included in each theme from phase two**

| **Offer a future** | **Facilitate good collaboration** | **Ensure connectedness** | **Provide a safe and stimulating environment** | **Ensure that (all) youth have the power to influence** | **Redress urban-centred images and policies** |
| --- | --- | --- | --- | --- | --- |
| - Make rural places attractive and liveable - More possibilities for internships and work in local companies - Offer activities that challenge gender stereotypes - Support to organizations that provide activities - Provide activities that are free of charge or affordable - Youth clinics that is open during weekends - More resources to specialised psychiatric care - Emergency contraception in supermarkets - Make it possible to continue studying in rural areas - Change the educational grading system | - Actors developing common objectives, procedures and structure - Ensure that agreements that are not dependent on individuals | - Make travel to city easier - Public transport adapted to local needs - 100% access to (good) internet connection | - Street lights and well-cleared forest areas - Childhood free from harassments, bullying and porn. - Permissive sports facilities, parks and schools - Mentally stimulating climate | - Help young people to “just be” - Ask young people what they need - LGBTQI+ skills for everyone who gets in contact with youth - Enhance knowledge among young people about rights and obligations - Stronger interventions for socially vulnerable individuals - Authorities should work more actively with young people - Youth ambassadors who are involved in decision-making processes | - Promote the image of a positive future in rural places - Stop privatization processes - Problematize the right to choose a school - Take advantage of influencers who provide a positive image of rural places |

**Supplementary table 3. Examples of strategies included in each cluster from phase three with mean scores on the feasibility (f) and importance (i) ratings**

| **Guarantee an accessible and sustainable health care services’ network** | **Enhance knowledge about and competence on critical issues** | **Provide relevant education of good quality** | **Be attractive and lively** | **Assure youth-centred approaches and equity** |
| --- | --- | --- | --- | --- |
| - Ensure that care reaches inaccessible places   (f 2.22; i 4.63)   - More flexibility and time in consultations with young people   (f 3.06; i 4.42)   - Mobile youth clinics "on tour"   (f 3.11; i 3.63)   - Care and treatment programs adapted to local conditions and needs   (f 3.11; i 4.21)   - Develop a first-line specialised psychiatry care   (f 2.67; i 4.16)   - Increase knowledge about youth clinics and their role   (f 4.05; i 4.05)   - Take youth problems and ill health seriously   (f 3.89; i 4.05) | - Offer parent groups and support programs for parents   (f 3.63; i 4.46)   - Create a climate that promotes help seeking for mental ill health   (f 3.26; i 4.53)   - Ensure that staff have cultural competence   (f 3.61; i 4.32)   - Make sure that girls exposed to violence and harassment have access to support   (f 3.68; i 4.74)   - Critically discuss masculinity and femininity norms   (f 3.84; i 4.74)   - Support youth with a norm-breaking functionality   (f 3.28; i 4.16) | - Increase the possibility of distance learning   (f 3.79; i 4.11)   - Offer the program YAM (youth aware of mental health) in all schools   (f 3.47; i 3.74)   - Strengthen school health services   (f 3.21; i 4.32)   - Increase the resources for and the number of qualified teachers in schools   (f 2.79; i 4.32)   - Lower knowledge requirements and focus on in-depth learning in school   (f 2.42; i 3.11)   - Structures for prioritizing health promotion in schools   (f 3.63; i 3.95)   - Helping and supporting ‘NEETs’   (f 3.11; i 4.37) | - Provide high quality broadband to everyone   (f 3.26; i 4.05)   - Strengthen young people's entrepreneurship   (f 3.37; i 3.84)   - Establish facilities with activities that can meet different needs of young people   (f 2.84; i 3.89)   - Create and offer an inclusive cultural life   (f 3.32; i 4.21)   - Maintain and strengthen the sense of community in rural areas   (f 3.37; i 4.37) | - Implement the human rights of indigenous peoples, including Sámi youth's right to identity, culture, language, country and history as a basis for their own health, in all public activities   (f 3.05; i 4.16)   - Promote multilingualism, especially in all the national minority languages   (f 3.16; i 3.89)   - Empathic encounters where the needs of the young people are at the centre   (f 3.95; i 4.47)   - Follow and implement the Convention on the Rights of the Child   (f 3.37; i 4.63)   - Prevent further privatization of service important to young people   (f 3.16; i 3.68) |
